# LncRNA RPARP-AS1 promotes the progression of osteosarcoma cells through regulating lipid metabolism

**DOI:** 10.1186/s12885-024-11901-x

**Published:** 2024-02-02

**Authors:** Feng Cai, Luhua Liu, Yuan Bo, Wenjing Yan, Xuchang Tao, Yuanxiang Peng, Zhiping Zhang, Qi Liao, Yangyan Yi

**Affiliations:** 1 The Third Affiliated Hospital of Nanchang University, North 128 Xiangshan Road, Nanchang, Jiangxi 330008 P.R. China; 2 Department of Orthopedics, The First Hospital of Nanchang, North 128 Xiangshan Road, Nanchang, Jiangxi 330008 P.R. China; 3 Department of Plastic Surgery, The Second Affiliated Hospital of Nanchang University, No. 1, Minde Road, Nanchang, Jiangxi 330008 P.R. China

**Keywords:** LncRNA RPARP-AS1, Lipid metabolism, Osteosarcoma, Gene set enrichment analysis, Akt/mTOR pathway

## Abstract

Osteosarcoma (OS) is a highly malignant tumor, and its dysregulated lipid metabolism is associated with tumorigenesis and unfavorable prognosis. Interestingly, long noncoding RNAs (lncRNAs) have emerged as pivotal regulators of lipid metabolism, exerting notable impacts on tumor proliferation. Nevertheless, the involvement of RPARP-AS1, a novel lipid metabolism-associated lncRNA, remains unexplored in the context of OS. This study aims to identify functionally relevant lncRNAs impacting OS proliferation and lipid metabolism and seeks to shed light on the upstream regulatory mechanisms governing lipogenic enzyme activity. Based on comprehensive bioinformatic analysis and the establishment of a risk model, we identified seven lncRNAs significantly associated with clinical characteristics and lipid metabolism-related genes in patients with OS. Among these, RPARP-AS1 was selected for in-depth investigation regarding its roles in OS proliferation and lipid metabolism. Experimental techniques including RT-qPCR, Western blot, cell viability assay, assessment, and quantification of free fatty acids (FFAs) and triglycerides (TGs) were utilized to elucidate the functional significance of RPARP-AS1 in OS cells and validate its effects on lipid metabolism. Manipulation of RPARP-AS1 expression via ectopic expression or siRNA-mediated knockdown led to alterations in epithelial-mesenchymal transition (EMT) and expression of apoptosis-associated proteins, thereby influencing OS cell proliferation and apoptosis. Mechanistically, RPARP-AS1 was found to augment the expression of key lipogenic enzymes (FABP4, MAGL, and SCD1) and potentially modulate the Akt/mTOR pathway, thereby contributing to lipid metabolism (involving alterations in FFA and TG levels) in OS cells. Collectively, our findings establish RPARP-AS1 as a novel oncogene in OS cells and suggest its role in fostering tumor growth through the enhancement of lipid metabolism.

**Supplementary Information:**

The online version contains supplementary material available at 10.1186/s12885-024-11901-x.

## Introduction

Osteosarcoma (OS) is a primary bone tumor that predominantly affects the adolescent and pediatric population [1]. Notably, OS frequently progresses to form pulmonary metastasis, which has historically posed significant therapeutic challenges due to the elevated metastasis rate and limited understanding of the pertinent molecular and functional mechanisms [2]. Despite the current treatment approach that involves a combination of surgical intervention, adjuvant chemotherapy, and radiotherapy leading to facilitated patient survival rates, the overall survival duration for individuals with OS remains unfavorable, typically falling below five years [3]. The emergence of novel therapeutic concepts and advancements in molecular targets offers the potential to augment the prognosis for patients with OS. Hence, it is of significant importance to conduct comprehensive investigations into the pathogenesis of OS to advance diagnostic and treatment modalities. The conversion from oxidative phosphorylation (OXPHOS) to glycolysis has been recognized to underpin the characteristics of OS [4], thereby implicating that metabolic alterations in OS cells are pivotal contributors to its occurrence and unfavorable prognostic outcomes [5]. Consequently, delving into the metabolic processes associated with OS can facilitate the elucidation of the underpinnings driving its development.

The metabolic profile of cancer is commonly characterized by metabolic demands, nutrient supply, and the regulatory influence of metabolic enzymes [6]. Essential nutrients, such as glucose, amino acids, and fatty acids, support the heightened energy demands of tumor cells, thereby fostering malignancy with high proliferative and invasive capabilities [7]. A growing body of research suggests that the interplay between metabolic regulation and genetic factors impart significant effects on cancerigenesis and tumor progression [8]. Therefore, the specific targeting of tumor metabolism is gaining recognition as a novel and promising therapeutic strategy. Tumor metabolism typically involves glucose, glutamine, and lipid metabolism [7]. Prior investigations have confirmed the vital role of lipid metabolism in facilitating tumor cell growth and proliferative capability, thus supporting the heightened energy demand of tumor cells [9, 10]. Moreover, mounting clinical and laboratory data have unveiled the critical role of lipid metabolic dysfunction in tumorigenesis, tumor progression, and treatment outcomes [11, 12]. To date, several risk assessment models have been developed to delineate the prognostic value of genes related to tumor microenvironment (TME), immune cell infiltration, and energy metabolism in the context of OS [13, 14]. Nonetheless, there is a paucity of research addressing the modulation of lipid metabolism by long noncoding RNAs (lncRNAs) in OS. The exploration into the molecular mechanisms through which lncRNAs modulate lipid metabolism holds the potential to establish a theoretical foundation for the development of tumor-targeted therapeutic strategies.

LncRNAs have been established as nucleotide transcripts over 200 bp in length, with no protein-coding capacity [15]. Historically, lncRNAs were widely considered as biologically inert and functionally irrelevant genomic components, often designated as residual DNA material in cellular contexts. However, a substantial body of research has demonstrated that lncRNAs frequently serve as molecular decoys, effectively sequestering microRNAs (miRNAs or miRs) to modulate the expression of genes involved in glycolysis, lipid metabolism, and various signaling pathways. The capacity of lncRNAs to function as molecular sponges establishes their significance in cancer metabolism and tumorigenesis [16]. For example, prior research has demonstrated that certain lncRNAs can facilitate the metastatic potential or affect chemotherapy resistance in OS by sponging specific miRNAs [17]. Nonetheless, further investigation is warranted to fully unravel the mechanisms and impacts of lncRNA sponge activity across diverse cancer types. RPARP-AS1, also referred to as C10orf95-AS1, is a lncRNA with a length of 1622 bp, which is located in the genomic region Chr10:q24.32. Functionally, RPARP-AS1 has been established to encourage the proliferative, migratory, and invasive potential of colorectal cancer (CRC) cells by acting as a sponge for miR-125a-5p [18]. Additionally, the impact of RPARP-AS1 on prognosis has been documented in various solid tumors, such as triple-negative breast cancer, lung adenocarcinoma, and colon cancer [19–21]. However, insufficient evidence is available to support the validation of the role of RPARP-AS1 in orchestrating lipid metabolism and augmenting tumorigenesis in OS studies.

The development and utilization of risk assessment models assume a crucial role in the field of cancer research. The risk assessment models can pinpoint individuals or populations with an elevated risk of specific cancer types [22]. These models typically incorporate data related to age, family history, environmental exposure, and lifestyle factors. Furthermore, genetic screening techniques can be utilized to identify genetic variations associated with cancer development. The involvement of the Akt/mTOR pathway has been extensively explored in OS research. Overactivation of Akt has been found to trigger the downstream mTOR activation, thereby leading to rapid proliferation of tumor cells, increased secretion of cancer-associated proteins, expedited cell cycle entry, and shortened G1 phase arrest. These collective molecular processes create an environment fostering the rapid initiation and development of tumors [23].

Furthermore, researchers have shifted their focus toward investigating the involvement of lipid metabolism enzymes in OS [24]. During the progression of tumors, the availability of nutrients in the TME undergoes dynamic alterations. This phenomenon leads tumor cells to depend on lipid metabolism to fuel their rapid activities, including proliferation, survival, migration, invasion, and metastasis. These findings hold potential implications for the advancement of anti-cancer treatment.

In this study, we uncovered the role of RPARP-AS1 in affecting the proliferative and invasive capacities and the lipid metabolism of OS cells. These findings were established through a comprehensive examination of bioinformatics databases and the application of relevant analytical tools. Our research findings indicated that the upregulation of RPARP-AS1 led to a notable enhancement of OS cell proliferation, which aligns with the predictions derived from our bioinformatic analysis. Mechanistically, the upregulation of RPARP-AS1 might influence the Akt/mTOR pathway, thereby facilitating the upregulation of the lipogenic enzymes. This heightened capacity for lipid metabolism in OS cells assumed a contributory role in tumorigenesis. Conversely, the siRNA-mediated repression of RPARP-AS1 yielded an opposite effect. Hence, our findings not only presented a viable approach for the identification of lncRNAs associated with lipid metabolism-related functions but also shed light on the underlying molecular mechanisms governing lipid metabolism in OS.

## Results

### Enrichment of lipid metabolism-related lncRNAs with prognostic significance in OS

**Fig. 1:**
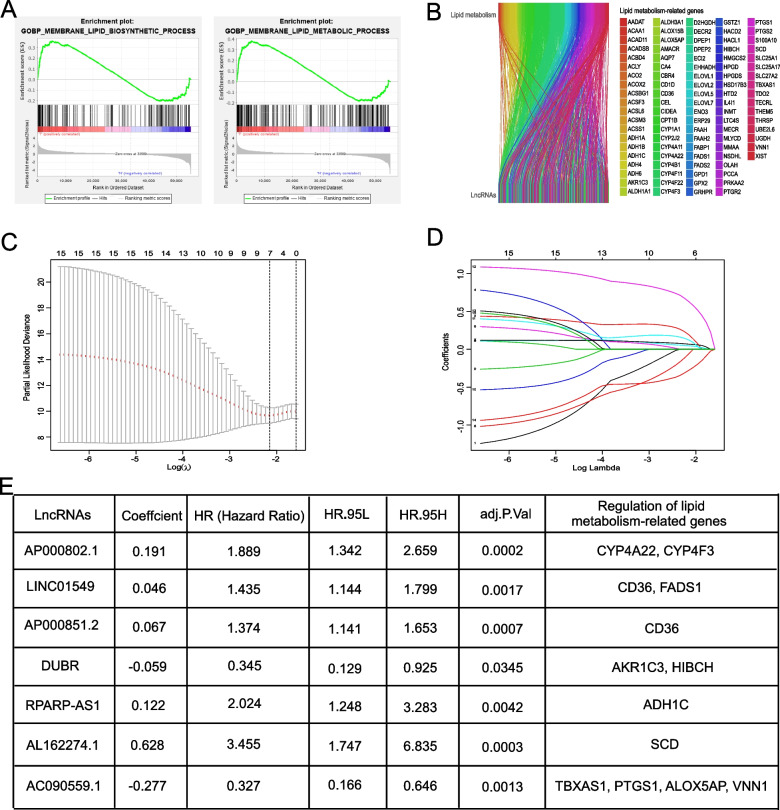
Construction of the risk prognostic models using lipid metabolism-related genes (LMRGs). **A** GSEA analysis revealed significant differences (*P* < 0.05) in the enrichment plots of LMRG sets between OS and normal tissues. **B** The correlation analysis diagram depicted the relationships between lipid metabolism-related mRNA (above) and lncRNAs (below), with varying shades of color representing different lipid metabolism-related mRNA associations. **C**-**E** Lipid metabolism-related lncRNAs and their respective regression coefficients in the prognostic model were determined through LASSO and multivariate and univariate Cox regression analyses

This study sought to investigate the key lncRNAs implicated in lipid metabolism in OS. To achieve this objective, we acquired transcriptomic data from 88 OS patients in the TCGA database and subjected this dataset to comprehensive bioinformatic analysis. Initially, we selected two canonical gene sets with established associations with lipid metabolism from the Molecular Signature Database (MSigDB). Subsequently, we employed gene set enrichment analysis (GSEA) to assess the enrichment of these gene sets, as illustrated in Fig. 1A, B. This analysis allowed us to pinpoint gene sets that exhibited a significant association with lipid metabolism. Following this enrichment analysis, we proceeded to conduct a differential analysis on these identified gene sets. The "ggalluvial" package was utilized to effectively visualize and elucidate the connections between the differentially expressed genes (DEGs) related to lipid metabolism and specific lncRNAs. Accordingly, a Sankey diagram (Fig. 1B) was generated, which unveiled the strong connections between specific lncRNAs and the DEGs related to lipid metabolism.

To delve deeper into the association between these identified genes and the progression of OS, we conducted univariate and multivariate COX regression as well as LASSO regression analyses based on the differential expression data. This analysis pinpointed seven significant lncRNAs associated with OS development: AP000802.1, LINC01549, AP000851.2, DUBR, RPARP-AS1, AL162274.1, and AC090559.1 (Fig. 1C and D). Considering the aforementioned findings, we advanced our research by constructing precise prognostic risk models based on multivariate COX regression analysis, incorporating the initially screened lncRNAs. The risk model was established using the following formula: 0.191 AP000802.1 + 0.046 LINC01549 + 0.067 AP000851.2 + (-0.059 DUBR) + 0.122 RPARP-AS1 + 0.628 AL162274.1 + (-0.277 AC090559.1) (Fig. 1E).

### Clinical prognostic value of the identified lipid metabolism-related lncRNAs based on risk models

**Fig. 2:**
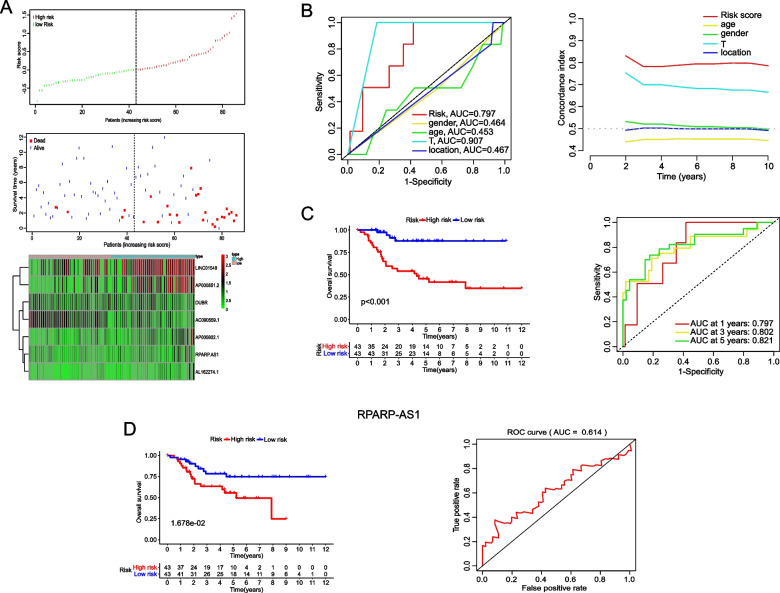
Analysis of risk signatures. **A** Association between survival status and risk scores, as well as the expression of the seven lncRNA risk factors, in OS patients. Data were ranked from low to high based on moderate scores. **B** ROC curve analysis and univariate and multivariate Cox regression analysis of the lipid metabolism risk model and the related clinical characteristics. **C**, **D** Kaplan–Meier survival analysis and ROC curve analysis for high-risk and low-risk groups **C** and RPARP-AS1 risk factors **D**

Subsequently, we sought to validate the prognostic clinical value of the constructed risk model in patients with OS. Initially, the patients were categorized into a high-risk group (HRG) and a low-risk group (LRG) based on the median risk score to calculate risk scores for each OS patient using the established risk formula (Fig. 2A). As illustrated, the HRG exhibited a markedly elevated mortality rate compared to the LRG, and the proportion of prognostic risk attributed to the seven lncRNAs was notably greater in the HRG than in the LRG. Therefore, we considered the integration of the lipid metabolism risk score with clinical prognostic indicators to assess the clinical significance of all these lncRNAs. Univariate and multivariate regression analyses were performed on various clinical characteristics, including the established lipid metabolism risk score. Significant differences and correlations were observed in clinical characteristics of the constructed lipid metabolism-related lncRNA risk factors. These factors could serve as independent prognostic indicators for OS patients, as supported by a substantial area under the curve (AUC) of 0.797 and a high concordance index of 0.85 (Fig. 2B). Following this, a Kaplan–Meier (KM) analysis was conducted, leading us to determine that the overall survival rate was notably lower in the HRG than the LRG (*P* < 0.001). Furthermore, receiver operating characteristic (ROC) curve analysis confirmed the accuracy of the risk model (AUC = 0.797) (Fig. 2C). Survival and ROC curves for seven lncRNAs were presented in Fig. 2D and Supplemental Figure S1, respectively. The analysis uncovered that the AUC values of the ROC curves for DUBR (AUC = 0.361) and AC090559.1 (AUC = 0.294) were below 0.5, indicating their poor accuracy in predicting outcomes. Conversely, RPARP-AS1 (AUC = 0.614), AP000802.1 (AUC = 0.634), LINC01549 (AUC = 0.723), AP000851.2 (AUC = 0.6), and AL162274.1 (AUC = 0.764) exhibited AUC values exceeding 0.5, implying comparatively better predictive accuracy. Therefore, due to the inaccurate survival curve results observed in Fig. 2D and Supplementary Figure S1A, DUBR and AC090559.1 were excluded from subsequent analyses.

Additionally, the expression of the remaining five lncRNAs in cancerous and adjacent non-cancerous tissues of OS patients. The analysis revealed that only RPARP-AS1 and AP000802.1 exhibited significant expression differences between the cancerous and adjacent non-cancerous tissues (**P < 0.01) (Supplementary Figure S2A). Based on these findings, knockdown experiments targeting these two lncRNAs were conducted in MG63 cells, followed by CCK8 assays to evaluate cell viability. Interestingly, the results indicated that the silencing of AP000802.1 imparted no notable effects on the viability of OS cells (n.s. = not significant) (Supplementary Figure S2B and C). Consequently, the focus of the study was shifted to RPARP-AS1 for further investigation. Additional research using the LncBook 2.0 database identified RPARP-AS1 as a novel transcript located on the antisense strand of chromosome 10:102,435,733–102461106 (Supplementary Figure S2D). These findings provide strong support for the significant anti-cancer effect of RPARP-AS1 and its potential clinical importance.

### Silencing of RPARP-AS1 diminished the proliferation and fostered the apoptosis of OS cells

**Fig. 3:**
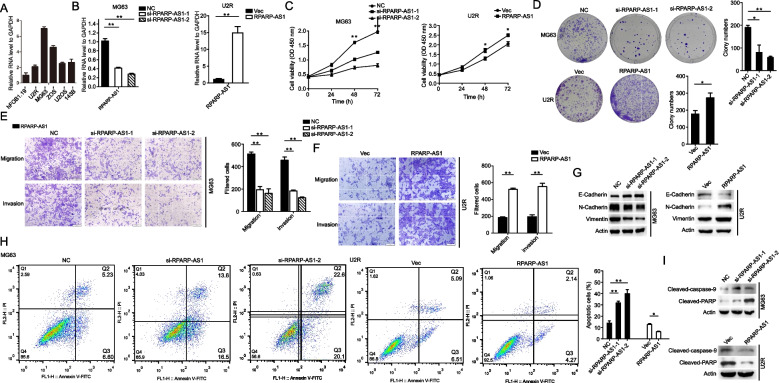
RPARP-AS1 facilitated the proliferation and migration of OS cells. **A** The mRNA expression of RPARP-AS1 in five OS cell lines and the normal cell line. **B** MG63 or U2R cells were transfected with si-NC, si-RPARP-AS-1, si-RPARP-AS1-2, empty vector, or pcDNA3.1-RPARP-AS1 for 72 or 48 h. The efficiency of silencing or overexpression of RPARP-AS1 was assessed via qRT-PCR in MG63 or U2R cells, respectively. **C** Measurement of cell viability (MG63 and U2R cells) by CCK8 assay after silencing or overexpression of RPARP-AS1 for 72 or 48 h. **D**-**F** Results of cells colony formation, migration, and invasion assays after silencing or overexpression of RPARP-AS1 in MG63 or U2R cells, respectively. After 24 h of silencing or overexpression of RPARP-AS1, cells were resuspended and seeded into 6-well plates (1,000 cells/well) (**D**) or 12-well plates (8 × 10^4^ or 5 × 10^4^ cells/well) (**E** and **F**) for the indicated time. Scale bar, 100 μm. **G** Protein levels of RPARP-AS1, E-cadherin, N-cadherin, and vimentin were determined by Western blot analysis after silencing or overexpression of RPARP-AS1. **H**, **I** Cell apoptosis was assessed by flow cytometry (**H**) and Western blot analysis (**I**) after silencing or overexpression of RPARP-AS1 in MG63 or U2R cells. In **B**-**F** and **H**, a two-tailed unpaired Student’s t-test was used to determine statistical significance, with GAPDH or β-actin used as a loading control. Data are presented as mean ± SD (*n* = 3). *P < 0.05, **P < 0.01, compared to NC or Vector

In light of the predicted high expression of RPARP-AS1 in OS from database analyses, we examined the mRNA levels of RPARP-AS1 in five OS cell lines and the normal cell lines (Fig. 3A). Following this, siRNAs were synthesized, and their knockdown efficiency was validated in MG63 cells, which exhibited high RPARP-AS1 expression. Conversely, the efficiency of overexpression was assessed in U2R cells, which showed low RPARP-AS1 expression. The introduction of si-RPARP-AS1-1 and si-RPARP-AS1-2 significantly diminished RPARP-AS1 levels in MG63 cells (**P < 0.01), while the use of pcDNA3.1-RPARP-AS1 overexpression plasmids markedly elevated the mRNA levels of RPARP-AS1 in U2R cells (**P < 0.01) (Fig. 3B). Further analysis, as indicated in Fig. 1E, demonstrated that RPARP-AS1 modulated the expression of the lipid metabolism-related gene (LMRG) ADH1C. This finding was corroborated by results showing that knockdown or overexpression of RPARP-AS1 notably elevated or diminished the mRNA level of ADH1C (*P < 0.05 and **P < 0.01), as depicted in Supplementary Figure S2E. To validate the predictions from the database, we performed a series of experiments including CCK8, colony formation, transwell, and apoptosis assays. The results unveiled that siRNA-mediated targeting of RPARP-AS1 notably diminished the proliferative, migratory, and invasive capacities of MG63 cells, while ectopic expression of RPARP-AS1 produced contrary effects in U2R cells (*P < 0.05 and **P < 0.01), as presented in Fig. 3C-F. Given the strong association between tumorigenesis and epithelial-mesenchymal transition (EMT), it was hypothesized that RPARP-AS1 might influence the expression of EMT-related proteins. This hypothesis was confirmed by observations of altered RPARP-AS1 levels being accompanied by changes in the expression of E-cadherin, N-cadherin, and vimentin (Fig. 3G). It was also noted that RPARP-AS1 knockdown led to increased cell death, a phenomenon supported by the data presented in Fig. 3H. The introduction of si-RPARP-AS1-1 and si-RPARP-AS1-2 augmented early and late apoptosis and elevated the protein levels of cleaved caspase-9 and PARP compared to control groups. In contrast, overexpression of RPARP-AS1 diminished apoptosis and levels of cleaved caspase-9 and PARP (*P < 0.05 and **P < 0.01), as depicted in Fig. 3H and I. These results indicate RPARP-AS1 as a functional gene capable of affecting OS cell proliferation through mechanisms involving EMT and apoptosis.

### RPARP-AS1 enhanced lipid metabolism in OS cells by upregulating the expression of lipogenic enzymes and influencing the Akt/mTOR pathway

**Fig. 4:**
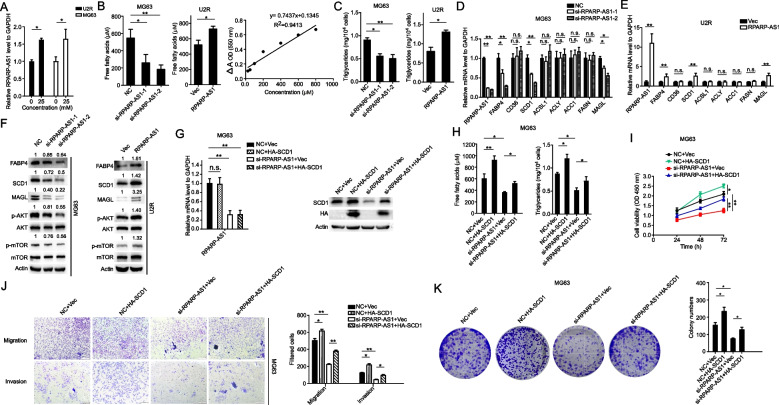
RPARP-AS1 elevated the lipid content and lipogenic enzyme expression in OS cells. **A** U2R and MG63 cells were exposed to various concentrations of glucose for 12 h after 50 min of starvation, and the results were determined by qRT-PCR and normalized to the 0 mM concentration. **B**, **C** Intracellular levels of FFAs (**B**, OD = 550 nm) and TGs (**C**, OD = 420 nm) were determined in OS cells in response to silencing (MG63) and overexpression (U2R) of RPARP-AS1. **D**, **E** Real-time PCR assay revealed the expression of FABP4, CD36, SCD1, ACSL1, ACLY, ACC1, FASN, and MAGL in MG63 or U2R cells stably transfected with si-NC, si-RPARP-AS1-1, si-RPARP-AS1-2 (**D**) or Vector, RPARP-AS1 (**E**). **F** Detection of the expression of lipogenic enzymes and the Akt/mTOR pathway-related proteins in MG63 or U2R cells after silencing or overexpression of RPARP-AS1. The levels of the indicated proteins were determined by Western blot. **G** Overexpression of SCD1 after silencing of RPARP-AS1. MG63 cells were transfected with pooled siRNAs containing two siRNAs for 24 h and then ectopically HA-tagged SCD1 for another 24 h. The mRNA and protein levels of RPARP-AS1 and SCD1 were detected by qRT-PCR and Western blot. **H** Ectopic expression of SCD1 counteracted the si-RPARP-AS1-mediated downregulation of FFAs and TGs. After silencing of RPARP-AS1 for 24 h, the MG63 cells were subsequently subjected to SCD1 overexpression. After 48 h, intracellular levels of FFAs and TGs were assayed using the respective assay kits. (I-K) Ectopic expression of SCD1 rescued the si-RPARP-AS1-mediated inhibition of MG63 cell proliferation, migration, and invasion. After silencing of RPARP-AS1 for 24 h, the MG63 cells underwent SCD1 overexpression. Then, cell proliferation, migration, and invasion were assayed by CCK8 assay after 72 h of SCD1 overexpression (**I**), or suspending cells into 6-well plates or 24-well plates for migration and invasion assay (**J**), colony formation assay (**K**) after 24 h of SCD1 overexpression. Scale bar, 100 μm. In A-E and **G**-**K**, a two-tailed unpaired Student’s t-test was used to determine statistical significance, with GAPDH or β-actin used as a loading control. Data are presented as mean ± SD (*n* = 3). *P < 0.05, **P < 0.01, n.s. = not significant, compared to NC or Vector

Lipid metabolism is essential for fulfilling the high energy requirements ofthe tumor proliferation. From above-mentioned bioinformatics analyses, RPARP-AS1 was pinpointed as a lncRNA potentially associated with lipid metabolism. This finding led to the investigation of the role of RPARP-AS1 in influencing lipid metabolism in OS cells. Considering the critical role of glucose as a carbon source for lipid synthesis [25], the study explored the effect of glucose at varying concentrations on RPARP-AS1 expression. As depicted in Fig. 4A, cells exposed to glucose at a high concentration (25 mM) exhibited increased expression of RPARP-AS1 compared to those treated with 0 mM glucose (*P < 0.05). This observation confirmed a potential association between RPARP-AS1 expression and lipid metabolism. Furthermore, we substantiated the ability of RPARP-AS1 to impact lipid metabolism in OS cells by measuring intracellular levels of free fatty acids (FFA) and triglycerides (TG) (*P < 0.05 and **P < 0.01) (Fig. 4B and C). Subsequently, the study examined whether RPARP-AS1 affected key enzymes involved in lipid metabolism. The results of qRT-PCR and Western blot demonstrated that RPARP-AS1 silencing or overexpression had regulatory effects on recognized lipid-metabolizing enzymes, such as FABP4, MAGL, and SCD1 (*P < 0.05 and **P < 0.01). However, no significant changes (n.s. = not significant) were observed in the mRNA levels of other lipid-metabolizing enzymes like CD36, ACSL1, ACLY, ACC1, and FASN (Fig. 4D-F).

Considering the essential regulatory role of the Akt/mTOR signaling pathway in lipid synthesis [26], we speculated that RPARP-AS1 might impact this pathway, thereby influencing lipid metabolism. Evidence from Fig. 4F indicated that overexpression of RPARP-AS1 facilitated the levels of phosphorylated (p-) Akt and p-mTOR, while silencing RPARP-AS1 yielded opposite effects (Fig. 4F). These results further suggested a potential role for RPARP-AS1 in modulating lipid metabolism in OS cells. The enzyme SCD1, which is responsible for converting saturated fatty acids (SFAs) into monounsaturated fatty acids (MUFAs), assumes a crucial role in lipid synthesis and tumor cell activities, thereby fostering cell proliferation [25, 27, 28]. In Fig. 4G, the introduction of SCD1 into MG63 cells counteracted the downregulation of SCD1 caused by si-RPARP-AS1 (**P < 0.01). Additionally, overexpression of SCD1 negated the inhibitory effect of RPARP-AS1 silencing on cell proliferation, accompanied by the restoration of intracellular levels of FFAs and TGs (*P < 0.05 and **P < 0.01) (Fig. 4H-K). As illustrated in Fig. 4I-K, overexpression of SCD1 rescued the cell viability, number, migration, and invasive abilities, which were compromised by RPARP-AS1 silencing. These findings imply that RPARP-AS1 may influence OS cell proliferation through the regulation of lipid metabolism.

### RPARP-AS1 accelerated tumor growth of OS cells in *vivo*

**Fig. 5:**
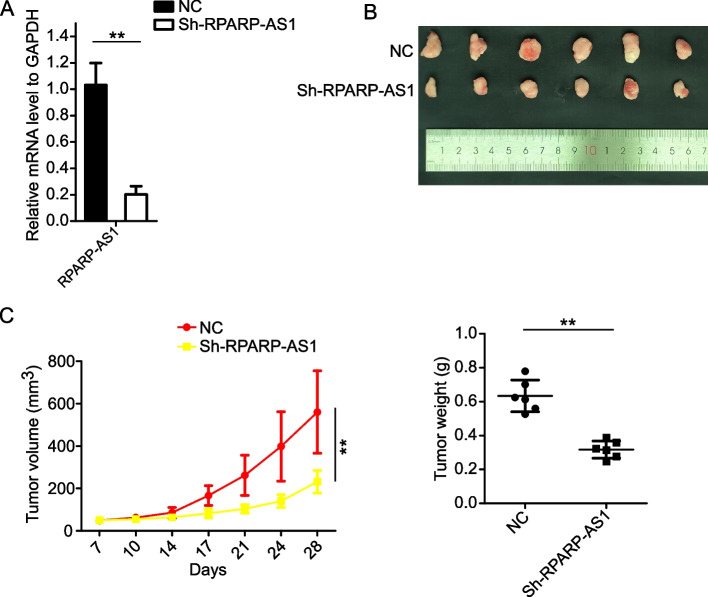
RPARP-AS1 augmented tumor growth in *vivo*. **A** RPARP-AS1 mRNA levels in MG63 cells with or without stable knockdown were determined using qRT-PCR. **B** Representative images of tumor-bearing BALB/c nude mice (*n* = 6). **C** Tumor growth curves and weights for each group as indicated in Fig. 5B. In A and C, a two-tailed unpaired Student’s t-test was used to determine statistical significance, with GAPDH serving as a loading control. Data presented as mean ± SD (*n* = 3 in Fig. 5A or *n* = 6 in Fig. 5C). **P < 0.01, compared to NC

In our in vitro experiments, it was established that RPARP-AS1 facilitated the proliferative potential of OS cells. A nude mouse model was developed to extend this investigation to an in vivo context. We successfully established the model by injecting sh-RPARP-AS1 MG63 cells, resulting in a stable knockdown of RPARP-AS1 (**P < 0.01) (Fig. 5A). Notably, the findings in vivo aligned with the in vitro results. The tumor volume and weight in the sh-RPARP-AS1 group were markedly diminished compared with the NC group (NC: 560.58 mm^3^ and 0.634 g; sh-RPARP-AS1: 231.83 mm^3^ and 0.318 g) (**P < 0.01) (Fig. 5B and C). These conclusive results provide evidence of the involvement of RPARP-AS1 in the progression of OS.

## Discussion

Recent research has provided evidence of the crucial role played by abnormal lipid metabolism in tumor progression [29, 30]. The intracellular lipid metabolism involves the conversion of TGs into absorbable long-chain fatty acids (LCFAs). Several key enzymes in lipid metabolism, along with the Akt/mTOR signaling pathway, are involved in subsequent processes such as lipid uptake (CD36, FABP4), lipolysis (MAGL, ACSL1) and lipid synthesis (ACLY, ACC1, FASN, SCD1) [31, 32]. In multiple tumor types, the overexpression of genes related to lipid uptake, lipid accumulation, and lipolysis has been linked to intensified proliferative and invasive potential and unfavorable prognosis [13, 33–38]. Several studies have illuminated the relationship between lipid metabolism and lncRNAs with a focus on genes such as FABP4, ACC1, SCD1, and FASN [29, 39–41]. Additionally, the activation of the Akt/mTOR pathway has been documented to upregulate the expression of SREB, which subsequently orchestrates ACC1 and ACLY and precipitates lipid synthesis [42–44]. Therefore, lipogenic enzymes and the Akt/mTOR pathway have become extensively studied targets in the mechanisms underlying lipid metabolism.

The upregulation of RPARP-AS1 expression in CRC and its association with unfavorable prognosis have been documented previously [18]. Interestingly, bioinformatic analyses disclosed an association of RPARP-AS1 with the TME, nonalcoholic fatty liver disease, and the occurrence and development of OS [45, 46]. These findings indicate that the expression of RPARP-AS1 is not limited to OS and may have implications in the tumorigenesis and prognostic outcomes of other tumor types. However, these associations have not been experimentally validated, leaving the relationship between lipid metabolism and RPARP-AS1 in the context of OS remains unexplored.

The present study was conducted with the objective of determining the clinical significance of RPARP-AS1, which was identified through bioinformatic analysis as a lipid metabolism-associated lncRNA that influences the proliferative potential of OS cells. Previous studies have highlighted ADH1C as an anti-cancer gene and its capability to curtail cell proliferation in CRC when overexpressed [47]. Additionally, accumulating studies have suggested a link between ADH1C and lipid metabolism, with downregulation of its expression predicting a poorer prognosis in patients [48–52]. Our findings implied that RPARP-AS1 inversely regulated the mRNA level of ADH1C, which aligns with its function in OS. Nonetheless, the specific regulatory mechanism and function of RPARP-AS1 warrant further comprehensive exploration. Moreover, our study demonstrated that both the ectopic expression and knockdown of RPARP-AS1 impacted the EMT, thereby altering the proliferative, invasive, and apoptotic potential of OS cells. To confirm the potential involvement of RPARP-AS1 in lipid metabolism, we manipulated glucose concentration in the cell culture medium and observed corresponding changes in RPARP-AS1 expression. Subsequent cellular-level investigations confirmed that modulating RPARP-AS1 levels could affect lipid metabolism in OS cells by orchestrating intracellular levels of FFAs and TGs.

Considering the involvement of various lipogenic enzymes in lipid metabolism, it was hypothesized that RPARP-AS1 might exert regulatory control over their expression. This hypothesis was substantiated through qRT-PCR and Western blot analyses, which revealed that both ectopic expression and siRNA-mediated knockdown of RPARP-AS1 notably altered the levels of key lipogenic enzymes (FABP4, MAGL, SCD1). Furthermore, the overexpression of RPARP-AS1 was observed to elevate the levels of p-Akt and p-mTOR, while silencing RPARP-AS1 produced the contrary effect. In vitro experiments further corroborated that RPARP-AS1 fostered the proliferative capacity of OS cells. Collectively, these findings highlighted that RPARP-AS1 potentially impacted lipid metabolism in OS by regulating lipogenic enzymes, ultimately contributing to the malignant phenotypes. This study provides novel insights into the mechanisms underlying the modulation of lipid metabolism in OS by lncRNAs.

This research delved into the relationship between the lncRNA RPARP-AS1 and OS using bioinformatic analysis, cell biology experiments, and other methodologies. The findings indicated an association between RPARP-AS1 expression and the prognosis of OS patients, as well as the ability of RPARP-AS1 to influence tumor proliferation through the regulation of lipid metabolism pathways in tumor cells. However, it is crucial to acknowledge that correlation does not equate to causation. This study faced challenges in completely ruling out the effects of other potential mechanisms, and there may be intermediate factors involved in the relationship between RPARP-AS1 and clinicopathological changes in OS. RPARP-AS1 might selectively influence specific lipid metabolic pathways in tumor cells, rather than exerting a universal effect on overall lipid metabolism. This study focused on some selected representative genes, and did not cover the entire spectrum of lipid metabolism. It is possible that other genes involved in lipid metabolism, which were not examined in this study, could also be under the regulatory influence of RPARP-AS1. Additionally, the mechanism through which RPARP-AS1 exerts control over downstream genes is likely to be complex. It predominantly involves the modulation of key upstream transcription factors or regulatory factors, thereby influencing specific downstream genes. It is also noteworthy that the degree of responsiveness to RPARP-AS1 regulation varies among different genes. Specific genes, such as FABP4, could be more prone to regulation through the involvement of RPARP-AS1. However, it is important to acknowledge certain limitations in this study, particularly in not adequately explaining this phenomenon. Consequently, this issue warrants further investigation in subsequent studies to comprehensively unravel the specific mechanisms underlying RPARP-AS1-mediated regulation of lipid metabolism in tumor cells.

To establish a definitive causal relationship between RPARP-AS1 and the development of OS, it is necessary to conduct further in-depth mechanistic studies using gene knockout animal models. This study was based on preliminary exploration of the role of RPARP-AS1 in OS. However, it has not yet fully clarified its involvement in OS progression, thereby indicating some limitations. In addition, future studies are required to assess the therapeutic potential of targeting RPARP-AS1 in OS models more thoroughly. It is essential to conduct preclinical peripheral blood tests to determine whether RPARP-AS1 levels could be indicative as a biomarker for the progression of OS. Future research should be directed towards applying the knowledge gained from the association between RPARP-AS1 and OS into clinical practices.

This study elucidates the underlying molecular mechanisms through which lncRNAs influence lipid metabolism in OS, thus establishing a theoretical foundation for the development of targeted therapeutic approaches for OS. Our findings substantiated that the lncRNA RPARP-AS1, recognized for its role in the modulation of lipid metabolism, fostered the proliferative potential of OS cells. We have provided a concise elucidation of the molecular mechanistic underpinnings. Specifically, RPARP-AS1 facilitated the upregulation of key genes such as FABP4, MAGL, and SCD1, thereby impacting the Akt/mTOR pathway. This cascade potentially enhanced lipid metabolism and fostered the proliferation of OS cells. Nonetheless, further extensive research is necessary to fully understand the specific molecular mechanisms of the regulation of RPARP-AS1 in lipid metabolism. The identification of miRNAs and downstream regulatory proteins involved in the interaction with the RPARP-AS1 sponge holds significant importance. Additionally, an investigation into the downstream target proteins of the Akt/mTOR pathway is critical for establishing the influence of RPARP-AS1 on the lipid metabolism pathway. Moreover, it is particularly particularly crucial to conduct blockade or inhibitor assays to establish a direct link between RPARP-AS1 and the proliferation of OS cells via lipid metabolism. These experiments may yield valuable insights into the functional role of RPARP-AS1 in intensifying OS development.

## Materials and methods

### Ethics approval

The research protocols were reviewed and approved by the Medical Ethics Committee of The First Hospital of Nanchang (Ethics Number: KY2022054). Informed consent was obtained from all participants included in the study. Additionally, this research adhered to the Animal Care Guidelines of The First Hospital of Nanchang.

### Data acquisition

The transcriptome data of 88 OS cases were retrieved from The Cancer Genome Atlas (TCGA) database (accessible at https://cancergenome.nih.gov/). Additionally, data pertaining to 396 normal bone tissue samples were obtained from the Genotype-Tissue Expression (GTEx) database (available at www.gtexportal.org). These two datasets were merged and underwent logarithmic transformation (log2 (x + 1)). Prior to comparative analyses, the expression data from both datasets were standardized to FPKM values. Basic details of lncRNAs, especially, RPARP-AS1, were retrieved from the LncBook 2.0 database [53].

### Enrichment analysis

The relationship between OS and lipid metabolism function was examined using GSEA to assess lipid metabolism enrichment across selected databases. The molecular characteristics from the MSigDB were utilized, which can be accessed and downloaded at https://www.gsea-msigdb.org/gsea/msigdb/ genesets.jsp. This analysis enabled the acquisition of classical sets of LMRGs, including gene sets like gobp_MT e_lipid_metabolic_process and gobp_MT lipid_biosynthetic_process. For each analysis, 1000 permutations were performed on these gene sets to facilitate the derivation of a standardized enrichment score (NES).

### Construction of the lipid metabolism-related prognostic model

The prognostic role of LMRGs was explored in this study. The expression differences in genes associated with lipid metabolism between 396 normal samples and 88 patient-derived OS samples were analyzed using the "Limma" package. Parameters such as cor.test mRNA and lncRNA related to lipid metabolism were employed for correlation analysis. Finally, seven lncRNAs related to lipid metabolism and prognosis were pinpointed through univariate and multivariate Cox regression analyses to determine the accurate risk signature formula [54]. The risk score formula was established as follows:

$$\text{Risk Score}=\sum\limits_{\text{i}=1}^{\textrm{n}}{\text{Coef}}_{\textrm{i}}\ast{\text{X}}_{\textrm{i}}{,}$$

Wherein, 'Coefi' represents the coefficient, and 'x_i_' denotes the expression of each selected gene.

### Cell culture and experimental protocols

Five human OS cell lines (U2R, U2OS, ZOS, ZOS, and 143B) were purchased from NCACC (Shanghai, China). These cells were cultured in DMEM (11,965,092, Thermo Fisher Scientific, Waltham, MA, USA) containing 10% FBS and incubated at a constant temperature in an incubator (Thermo Fisher Scientific HERA cell 150I) (5% CO_2_, 37℃). For the glucose stimulation experiments, U2R cells were initially incubated for 24 h. Subsequently, the medium was discarded, and the cells were washed with PBS. They were then subjected to a starvation period in glucose-free medium for 50 min, followed by treatment with a specified concentration of glucose for 12 h. Post-treatment, the cells were harvested for qRT-PCR analysis. All cell lines were subjected to short tandem repeat profiling and an incubation period of no more than two months.

### CCK8 assay

The CCK8 assay was utilized to evaluate cell viability subsequent to siRNA interference or overexpression experiments. Initially, the treated cells were harvested and resuspended to achieve a density of 2 × 10^4^ cells/well for 24-well plates or 5 × 10^4^ cells/well for 12-well plates. Triplicate wells were prepared for each experimental group. The cells in these wells were incubated to facilitate complete attachment and growth. Afterward, 50 μl of CCK8 solution (#K1018, APExBIO, USA) was added to each well, followed by gentle mixing, and then incubation at 37 °C for 45 min. After incubation, the absorbance at 450 nm was measured using a microplate reader. The optical density (OD450) value served as an indicator of cell proliferative activity. Cell growth curves were generated, and statistical analyses were performed to determine differences between groups. The entire experimental procedure was carried out with careful avoidance of light exposure and adherence to stringent time management.

### Colony formation assay

The clonogenic capacity of cells post-treatment was assessed using the colony formation assay. Initially, the treated cells were harvested after a 24-h incubation in 12-well plates. These cells were then resuspended and seeded into 6-well plates at a density of 1,000 cells per well, with three replicate wells for each experimental group. The 6-well plates were then incubated at 37 °C in a 5% CO_2_ humidified incubator for two weeks to facilitate colony formation. The culture medium was refreshed every three days during this incubation period. After the two-week incubation, the culture medium was discarded, and the cells were gently washed with PBS. The colonies were immobilized using 4% paraformaldehyde for 15 min and subsequently stained with crystal violet solution for 30 min at room temperature. The excess staining solution was gently rinsed off under running tap water. The plates were then left to air-dry overnight. Images of the stained colonies were captured, with three replicates from each treatment group being photographed. The number of colonies was statistically analyzed to determine the significance of the results.

### Migration and invasion assays

The transwell migration and invasion assays were performed to evaluate cell motility. For the invasion assay, the upper chambers (#0140, BD FALCON, USA) were pre-coated with Matrigel (#356234, BD Biosciences, USA) diluted at a 1:6 ratio with serum-free medium. This setup was incubated at 37 °C for 4–5 h to facilitate gelling. Subsequently, 8 × 10^4^ treated cells, suspended in serum-free medium, were seeded into the upper chambers in 24-well plates, with three replicate wells for each group. In the lower chambers, 600 μl of complete medium with 10% FBS was added as a chemoattractant. After a 24-h incubation at 37 °C, non-invading cells on the upper surface of the membrane were gently removed using a cotton swab. Cells that had invaded through the Matrigel and membrane to the lower surface were immobilized with 4% paraformaldehyde for 15 min and stained with 0.1% crystal violet for 30 min. The number of invaded cells was then captured and counted under a microscope. For the migration assay, 5 × 10^4^ treated cells, resuspended in serum-free medium, were seeded into the upper chambers without a Matrigel coating. The subsequent steps were the same as the invasion assay. After a 48-h of incubation, the cells that had migrated were imaged, immobilized, and stained. Images of the stained cells were taken, and random photographs were captured of colonies in at least three different microscope fields of view. The average number of cells from three replicates was counted for statistical significance analysis.

### Cell apoptosis assay

Apoptosis detection was performed using an Annexin V-FITC/PI double staining kit in adherence to the protocols provided by the manufacturer. After treatments involving either siRNA knockdown or overexpression in 12-well plates, cells were collected using EDTA-free trypsin and subsequently washed twice with cold PBS. The cell pellet was then resuspended in 300 μl of 1 × binding buffer. To this suspension, 3 μl of FITC-conjugated Annexin V and 3 μl of propidium iodide (PI) solution were added and gently mixed. The cells were incubated for 30 min at room temperature in light-restricted environment. After incubation, apoptosis assessment was conducted using flow cytometry with a BD FACSCalibur instrument (BD FACSCalibur, USA). The fluorescence intensity of FITC was measured at 518 nm, and that of PI was measured at 620 nm. Cells that did not exhibit staining for either Annexin V or PI were deemed viable. Those displaying positive staining for Annexin V but negative for PI were categorized as early apoptotic. Conversely, cells showing positive staining for both Annexin V and PI were recognized as being in late apoptosis or necrosis. The proportions of viable, early apoptotic, and late apoptotic cells were quantitatively determined for each sample.

### Real-time PCR

Following the guidelines provided by the manufacturer, total RNA was extracted using a TRIZOL reagent (#15,596,026, Invitrogen, USA). Subsequently, cDNA was synthesized utilizing the PrimeScript™ RT reagent kit (#RR036A, Takara, Dalian, China). PCR amplification was conducted with the SYBR Premix Ex Taq II kit (#RR420A, Takara) on the CFX96 Real-Time System (CFX96™ Real-Time system, Bio-Rad, USA). For normalization purposes, GAPDH served as the internal reference gene. The relative expression was quantified using the 2^−∆∆Ct^ method. The primers are listed in Supplementary Table S.

### Western blot

Cell lysis was initiated by incubating the cells with RIPA lysis buffer (with protease inhibitors) on ice for 30 min. Subsequently, the lysates underwent high-speed centrifugation at 14,000 rpm for 25 min at 4℃. The protein concentration in the supernatants was determined using a BCA protein quantification kit (#PC0020, Solarbio, China). Protein samples were separated on SDS‒PAGE, with agarose concentrations adjusted based on molecular weight. Following electrophoresis, the proteins were transferred onto PVDF membranes using an appropriate voltage, and then subjected to overnight incubation with primary antibodies at 4℃. Afterward, the secondary antibodies were cross-linked at room temperature for 1–2 h. Finally, an ECL kit (#PA112, TIANGEN, China) was used for detection. The primary antibodies used in this study included rabbit anti-SCD1 (#28,678–1-AP, Proteintech, China), rabbit anti-MAGL (#14,968–1-AP, Proteintech), rabbit anti-FABP4 (#12,802–1-AP, Proteintech), rabbit anti-E-cadherin (#20,874–1-AP, Proteintech), rabbit anti-N-cadherin (#22,018–1-AP, Proteintech), rabbit anti-vimentin (#10,366–1-AP, Proteintech), rabbit anti-Cleaved caspase-9 (#9509, Cell Signaling Technology, USA), rabbit anti-PARP (#9542, Cell Signaling Technology, USA), rabbit anti-p-Akt (#9271L, Cell Signaling Technology), rabbit anti-Akt (#4691L, Cell Signaling Technology, USA), rabbit anti-p-mTOR (#2971L, Cell Signaling Technology), rabbit anti- mTOR (#2972S, Cell Signaling Technology), and rabbit anti-β-actin (#20,536–1-AP, Proteintech).

### Cell transfection and establishment of stable cell lines

The siRNAs were purchased from GenePharma, and their sequences can be found in Supplementary Table S. The siRNA transfection was performed according to the instructions provided with the Lipofectamine RNAiMAX (#13778030, Thermo Fisher Scientific). To achieve the overexpression of RPARP-AS1, a pcDNA 3.1-RPARP-AS1 plasmid was synthesized by GeneCreate Biotech (Wuhan, China). For SCD1 overexpression, the complete SCD1 sequences were cloned into the HA-pcDNA 3.1 vector using BamHI and XhoI endonucleases. Plasmids were co-transfected into cells using Lipofectamine 2000 (#11668500, Thermo Fisher Scientific) and Opti-MEM (#31985070, Thermo Fisher Scientific). Cells were collected for Western blot or qRT-PCR analysis after 24 h. To establish stable cell lines, sh-RPARP-AS1 lentiviruses or NC (pLKO.1-RPARP-AS1 or pLKO.1) were co-transfected with packaging plasmids (psPAX2/pMD2.G) in 293 T cells. The viral fluid collected after 48 h was used to infect MG63 cells. Following 2 weeks of puromycin screening, the efficiency of RPARP-AS1 silencing was determined by qRT-PCR. The specific shRNA sequences are provided in Supplementary Table S.

### Quantification of FFAs and TGs

After the overexpression or silencing of RPARP-AS1, cells were harvested for the analysis of TGs (4 × 10^6^ cells) and FFA (2 × 10^6^ cells). Then, the cells were subjected to lysis using either RIPA lysis buffer or a Heptane/isopropanol solution (1:1) and sonicated for 1 min (at an intensity of 20%, with 2-s pulses and 1-s pauses). The resulting samples were then centrifuged at 12,000 × g and 4 °C for 20 min, and the supernatant was collected for testing. The levels of FFAs and TGs were quantified using an FFA Content Assay Kit (#BC0590, Solarbio, China) and a TG Content Assay Kit (#BC0620, Solarbio) following the protocols provided by the manufacturers.

### Xenograft model

Female BALB/c nude mice (aged 5 weeks) were obtained from the Shanghai Institutes for Biological Sciences, Chinese Academy of Sciences (Shanghai, China). These mice were acclimatized in a specific pathogen-free (SPF) environment for 2–3 days. OS cells (1.0 × 10^7^ cells), with or without stable silencing of RPARP-AS1, were resuspended in 200 µl of PBS and subcutaneously injected into the flanks of the nude mice. Each group consisted of 6 mice. Tumor size was monitored starting from 7 days post-injection, and changes in tumor volume were recorded every 2 to 3 days. Tumor volume was calculated using the formula V (mm^3^) = (L × S^2^)/2 (L, longest diameter; S, shortest diameter). After 28 days, the mice were euthanized by cervical dislocation under anesthesia, and the tumors were removed for subsequent analysis. All animal experiments were approved by the Medical Ethics Committee of The First Hospital of Nanchang (KY2022054).

### Statistical analysis

The data were subjected to statistical analysis using SPSS version 16.0 (SPSS, IL, USA) and GraphPad Prism 5 version 8.0.1 (GraphPad, CA, USA). Differential gene expression related to lipid metabolism between the tumor and normal groups in the database was analysed by the Wilcoxon test. Survival analysis for OS patients was performed using the KM method. The accuracy of the risk models and the survival curves based on the seven lncRNAs was assessed through ROC analysis. Differences between the two groups were analyzed using a two-tailed unpaired Student's t-test, with Welch's correction applied at a 95% confidence interval. All data were presented as the means ± SD and were obtained from three independent experimental replicates (*n* = 3). In this study, significance was defined as a p-value < 0.05.

### Supplementary Information

**Additional file 1: Supplementary Figure S1.** Kaplan-Meier survival and corresponding ROC curves for lncRNAs other than RPARP-AS1.

**Additional file 2: Supplementary Figure S2.** (A) The expression levels of AP000802.1, LINC01549, AP000851.2, RPARP-AS1, and AL162274.1 in both cancer and paracancer tissues of osteosarcoma patients were assessed (*n*=6). (B-C) MG63 cells were transfected with si-NC, si-AP000802.1-1, si- AP000802.1-2 respectively for 72 h. Silencing of AP000802.1 levels were assessed via qRT‒PCR (B) and detection of cell proliferation (MG63 cells) by CCK8 after silencing of AP000802.1 for 24, 48, 72 h (C). (D) Genomic location, basic information (obtained from LncBook 2.0 database) of RPARP-AS1 (HSALNG0080293). (E) Silencing or overexpression of RPARP-AS1 influenced mRNA levels of ADH1C. MG63 or U2R cells were transfected with si-NC, si-RPARP-AS-1, si-RPARP-AS1-2, empty vector or pcDNA3.1-RPARP-AS1 respectively for 72 or 48 h. Silencing and overexpression of RPARP-AS1 and ADH1C mRNA levels were assessed via qRT‒PCR. (F) Analyze the pan-cancer dataset in the UCSC database to study the single gene RPARP-AS1 across various cancer types. In A-C and E, a two-tailed unpaired Student’s t test was used to determine statistical significance, GAPDH or Actin was used as a loading control. Data are means ± SD (*n*=6 or *n*=3). *P < 0.05, **P < 0.01, n.s.=not significant. compared to NC, Vector or paracancer tissues.

**Additional file 3: Supplementary Table S.** Information for primers and siRNA, shRNA sequences.

**Additional file 4.**

## Data Availability

The data that support the findings of this study are available on request from the corresponding author. The data are not publicly available due to privacy or ethical restrictions.

## Abbreviations

LncRNA: Long non-coding RNA
OS: Osteosarcoma
qRT-PCR: Quantitative real-time polymerase chain reaction
EMT: Epithelial-mesenchymal transition
FFA: Free fatty acid
TG: Triglyceride
LMRG: Lipid metabolism-related gene
HRG: High-risk group
LRG: Low-risk group
GSEA: Gene set enrichment analysis
OXPHOS: Oxidative phosphorylation
MSigDB: Molecular Signature Database
TME: Tumor microenvironment
CRC: Colorectal cancer
