# Supplementary material for: LncRNA RPARP-AS1 promotes the progression of osteosarcoma cells through regulating lipid metabolism
Source: BMC Cancer. 2024 Feb 2;24:166. doi: 10.1186/s12885-024-11901-x (PMC10835925; doi:10.1186/s12885-024-11901-x)
Supplement: Supplementary file 1 — Additional file 1: Supplementary Figure S1. Kaplan-Meier survival and corresponding ROC curves for lncRNAs other than RPARP-AS1. [file 12885_2024_11901_MOESM1_ESM.docx]

**
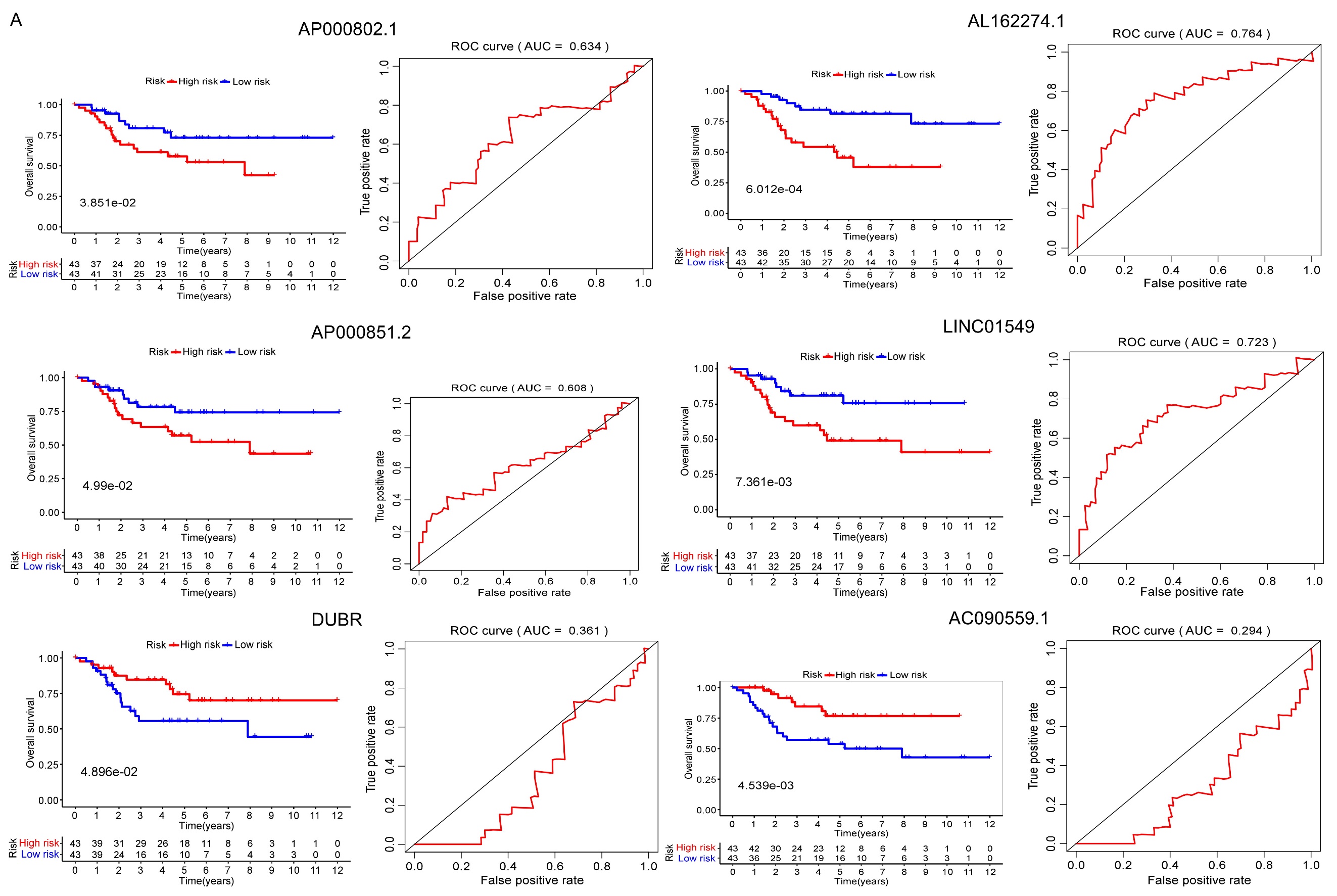
**

**Supplementary Figure S1.** **Kaplan-Meier survival and corresponding ROC curves for lncRNAs other than RPARP-AS1.** **(A)** Kaplan-Meier survival and ROC curve analysis of high and low-risk groups for six lipid metabolisms (AP000802.1, AL162274.1, AP000851.2, LINC01549, DUBR, AC090559.1) related lncRNAs risk factors.
