# Supplementary material for: LncRNA RPARP-AS1 promotes the progression of osteosarcoma cells through regulating lipid metabolism
Source: BMC Cancer. 2024 Feb 2;24:166. doi: 10.1186/s12885-024-11901-x (PMC10835925; doi:10.1186/s12885-024-11901-x)
Supplement: Supplementary file 2 — Additional file 2: Supplementary Figure S2. (A) The expression levels of AP000802.1, LINC01549, AP000851.2, RPARP-AS1, and AL162274.1 in both cancer and paracancer tissues of osteosarcoma patients were assessed (n=6). (B-C) MG63 cells were transfected with si-NC, si-AP000802.1-1, si- AP000802.1-2 respectively for 72 h. Silencing of AP000802.1 levels were assessed via qRT‒PCR (B) and detection of cell proliferation (MG63 cells) by CCK8 after silencing of AP000802.1 for 24, 48, 72 h (C). (D) Genomic location, basic information (obtained from LncBook 2.0 database) of RPARP-AS1 (HSALNG0080293). (E) Silencing or overexpression of RPARP-AS1 influenced mRNA levels of ADH1C. MG63 or U2R cells were transfected with si-NC, si-RPARP-AS-1, si-RPARP-AS1-2, empty vector or pcDNA3.1-RPARP-AS1 respectively for 72 or 48 h. Silencing and overexpression of RPARP-AS1 and ADH1C mRNA levels were assessed via qRT‒PCR. (F) Analyze the pan-cancer dataset in the UCSC database to study the single gene RPARP-AS1 across various cancer types. In A-C and E, a two-tailed unpaired Student’s t test was used to determine statistical significance, GAPDH or Actin was used as a loading control. Data are means ± SD (n=6 or n=3). *P < 0.05, **P < 0.01, n.s.=not significant. compared to NC, Vector or paracancer tissues. [file 12885_2024_11901_MOESM2_ESM.docx]

**
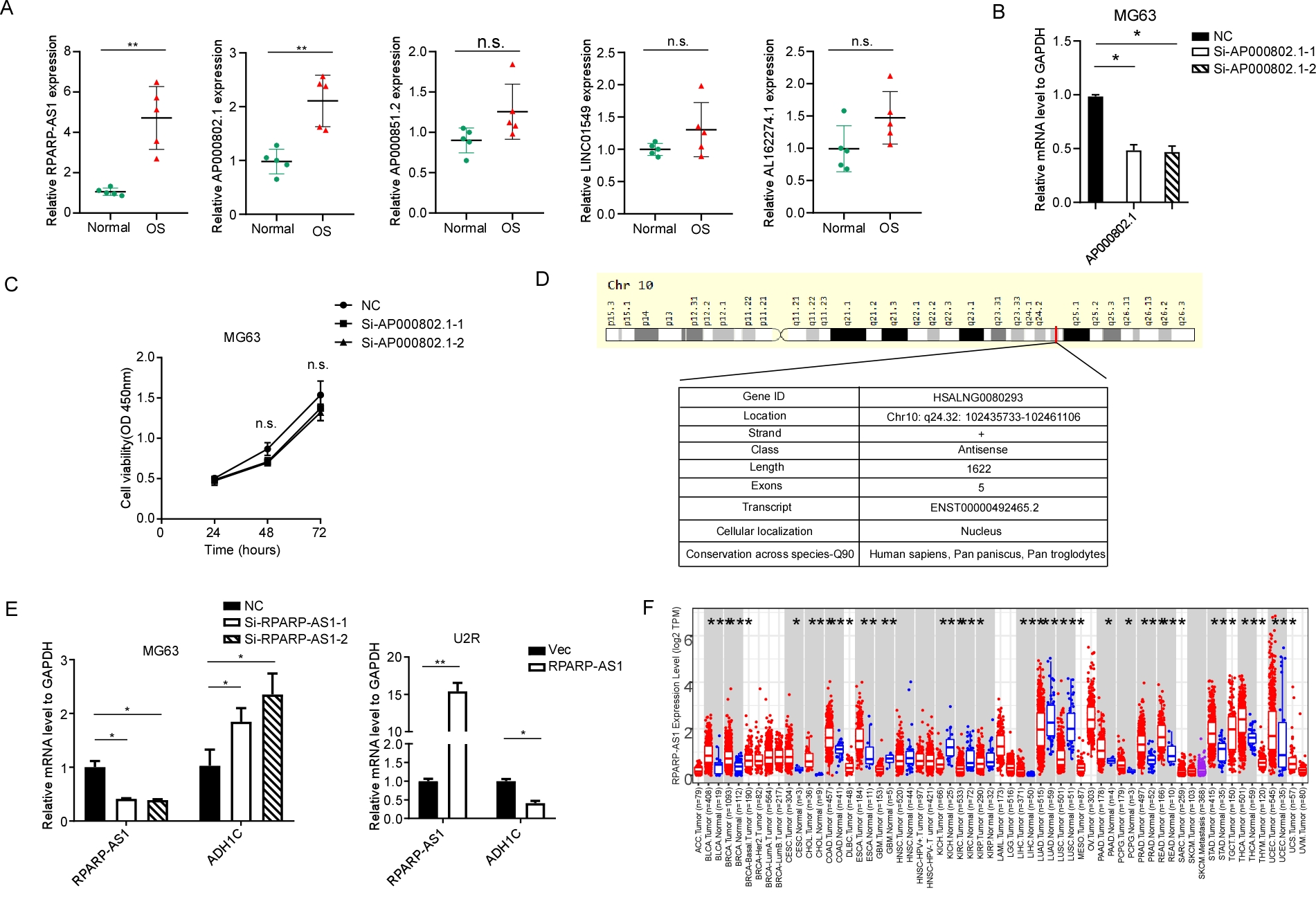
**

**Supplementary Figure S2.** **(A)** The expression levels of AP000802.1, LINC01549, AP000851.2, RPARP-AS1, and AL162274.1 in both cancer and paracancer tissues of osteosarcoma patients were assessed (n=6). **(B-C)** MG63 cells were transfected with si-NC, si-AP000802.1-1, si- AP000802.1-2 respectively for 72 h. Silencing of AP000802.1 levels were assessed via qRT‒PCR (B) and detection of cell proliferation (MG63 cells) by CCK8 after silencing of AP000802.1 for 24, 48, 72 h (C). **(D)** Genomic location, basic information (obtained from LncBook 2.0 database) of RPARP-AS1 (HSALNG0080293). **(E)** Silencing or overexpression of RPARP-AS1 influenced mRNA levels of ADH1C. MG63 or U2R cells were transfected with si-NC, si-RPARP-AS-1, si-RPARP-AS1-2, empty vector or pcDNA3.1-RPARP-AS1 respectively for 72 or 48 h. Silencing and overexpression of RPARP-AS1 and ADH1C mRNA levels were assessed via qRT‒PCR. **(F)** Analyze the pan-cancer dataset in the UCSC database to study the single gene RPARP-AS1 across various cancer types. In A-C and E, a two-tailed unpaired Student^’^s t test was used to determine statistical significance, GAPDH or Actin was used as a loading control. Data are means ± SD (n=6 or n=3). **P* < 0.05, ***P* < 0.01, n.s.=not significant. compared to NC, Vector or paracancer tissues.
