# Supplementary material for: LncRNA RPARP-AS1 promotes the progression of osteosarcoma cells through regulating lipid metabolism
Source: BMC Cancer. 2024 Feb 2;24:166. doi: 10.1186/s12885-024-11901-x (PMC10835925; doi:10.1186/s12885-024-11901-x)
Supplement: Supplementary file 3 — Additional file 3: Supplementary Table S. Information for primers and siRNA, shRNA sequences. [file 12885_2024_11901_MOESM3_ESM.docx]

**Supplementary Table S. Information for primers and siRNA, shRNA sequences**

| **Gene** | **Forward primer (5’-3’)** | | **Reverse primer (5’-3’)** |
| --- | --- | --- | --- |
| **qRT-PCR** | | | |
| RPARP-AS1 | GGCTCCTCTCCGCACTTTG | | AGGTGTAGACAGACGCTGGTA |
| AP000802.1 | AGGCCCGGATTCCGCGGCG | | GCAGGAAACAATTCGCCAAGAG |
| LINC01549 | AGCCTCGATCTCCTGGACTC | | CTGCCTTCTCCGTCACTCTG |
| AP000851.2 | CTTACCTGGTGCCTCCCTCCTC | | CCCTATTTGCCTCAGATTCATTCC |
| AL162274.1 | CGCTTCCCTGAGCAAAGGC | | TTGGCTAGATTCGCAAGACCAT |
| FABP4 | TGATGATCATGTTAGGTTTGGC | | TGGAAACTTGTCTCCAGTGAA |
| CD36 | AAAGTCACTGCGACATGATTAATGG | | AACGTCGGATTCAAATACAGCATAG |
| ACSL1 | GGTGGAAGTCACCAGCATGA | | CATTGCTCCTTTGGGGTTGC |
| SCD1 | AGCTCATCGTCTGTGGAGCC | | GCCACGTCGGGAATTATGAGG |
| ACLY | ATCGGTTCAAGTATGCTCGGG | | GACCAAGTTTTCCACGACGTT |
| ACC1 | AACAGCGTACAACACCGCCA | | GAGACCATTCCGCCCATCCG |
| FASN | ACAGCGGGGAATGGGTACT | | GACTGGTACAACGAGCGGAT |
| MAGL | CAAGGCCCTCATCTTTGTGT | | ACGTGGAAGTCAGACACTAC |
| GAPDH | GCACCGTCAAGGCTGAGAAC | | TGGTGAAGACGCCAGTGGA |
| **siRNA and shRNA targeting sequences** | | | |
| Si-RPARP-AS1-1 | | GCCUGGCCUGUGAUCAGAATT | |
| Si-RPARP-AS1-2 | | GCCAUCACCGGCUUUGAAUTT | |
| Si-AP000802.1-1 | | GUGACCACCUAGUCUCCAUTT | |
| Si-AP000802.1-2 | | CUGAAGGCUUGGGUAUGUATT | |
| pLKO.1-  -RPARP-AS1 | | CCGGGCCATCACCGGCTTTGAATCTCGAGATTCAAAGCCGGTGATGGCTTTTTTGGTACC | |
