# Supplementary material for: LncRNA RPARP-AS1 promotes the progression of osteosarcoma cells through regulating lipid metabolism
Source: BMC Cancer. 2024 Feb 2;24:166. doi: 10.1186/s12885-024-11901-x (PMC10835925; doi:10.1186/s12885-024-11901-x)
Supplement: Supplementary file 4 — Additional file 4. [file 12885_2024_11901_MOESM4_ESM.pdf]

Fig. 3G

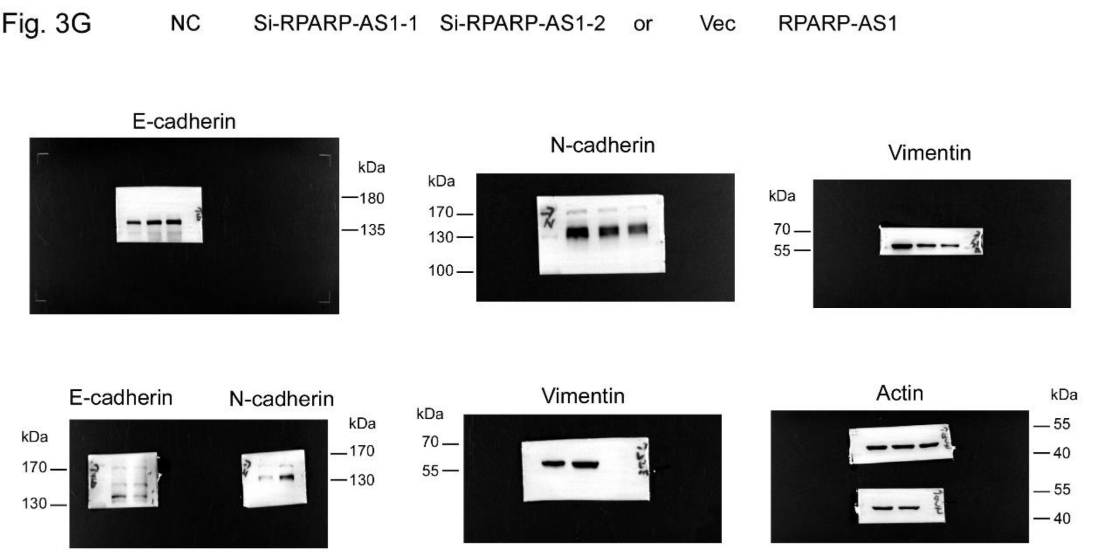

Fig. 3I

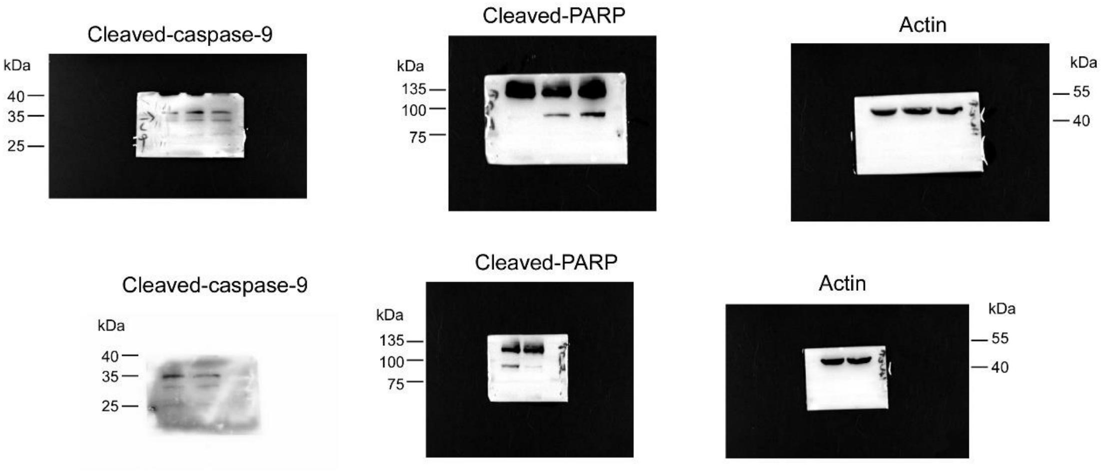

Fig. S1: Source data of western blot in Fig. 3G and 3I

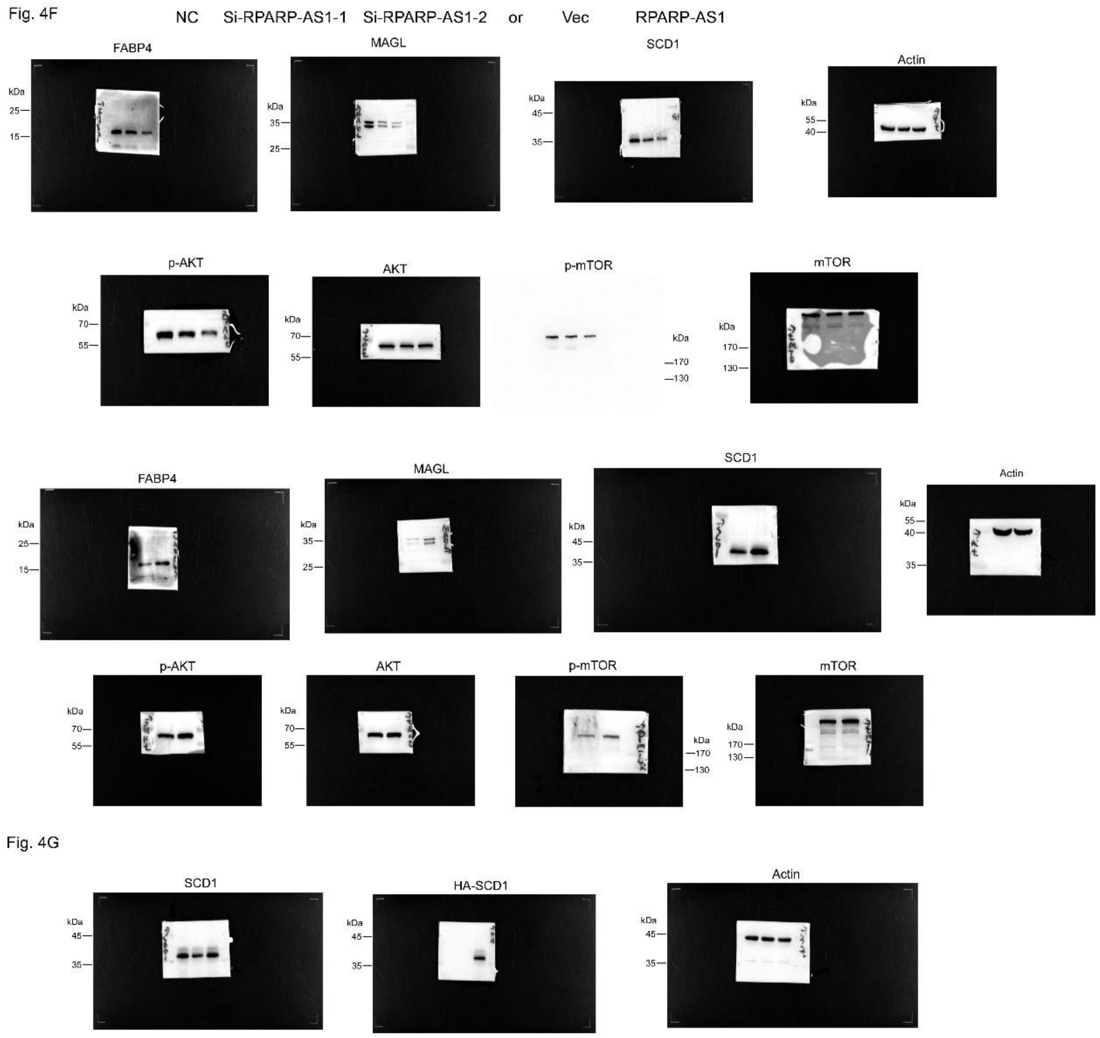

Fig. S2: Source data of western blot in Fig. 4F and 4G
